# Supplementary material for: Exploring the Nexus between Food Systems and the Global Syndemic among Children under Five Years of Age through the Complex Systems Approach
Source: Int J Environ Res Public Health. 2024 Jul 9;21(7):893. doi: 10.3390/ijerph21070893 (PMC11276875; doi:10.3390/ijerph21070893)
Supplement: Supplementary file 1 [file ijerph-21-00893-s001.zip › ijerph-3043433-supplementary.pdf]

Table S1. Experts' panel decisions on the connections between causes and consequences of the global syndemic.

Cause		Consequence	Decision of experts panel	Improvements at the meeting		
Low physical activity	-->	**Overweight**	exclusion due to lack of relevance to the study			
Low family income	-->	**Undernutrition**	improvement for clarification	Family's healthy eating practices, education, food access, public policies, and others	-->	Food purchase by family
Inadequate food consumption	-->	**Overweight and undernutrition**	approved			
Lack of social protection services and poor health access	-->	**Overweight and undernutrition**	exclusion due to lack of relevance to the study			
Inadequate family's food practice	-->	**Overweight and undernutrition**	improvement for clarification	Adequate eating habits by family	-->	Adequate eating habits by kids
Low parent's educational level	-->	**Overweight and undernutrition**	improvement for clarification	Family's healthy eating practices, education, food access, public policies, and others	-->	Food purchase by family
**Overweight and undernutrition**	-->	Mortality	approved			
**Overweight and undernutrition**	-->	Morbidity	approved			
Food production (animal production - high impact, plant production - low impact)	-->	**Climate change**	improvement for clarification	Livestock production	-->	**Climate change**
**Climate change**	-->	Food system	improvement for clarification	Extreme climate event	-->	Livestock production / fruit and vegetable production

**Climate change**	-->	Food-borne and other infectious diseases	exclusion due to lack of relevance to the study
**Climate change**	-->	Staple production	inclusion
Food purchase by school	-->	Food affordability	inclusion
Public policies, food access, and others	-->	Food purchase by school	inclusion
Livestock production	-->	Deforestation	inclusion
Food affordability	-->	Food purchase by school	inclusion
Food purchase by school	-->	Adequate eating habits by kids	inclusion
Climate change	-->	Extreme climate event	inclusion
Food purchase by family	-->	Food affordability	inclusion
Food purchase by family	-->	Adequate eating habits by family	inclusion
Deforestation	-->	Climate change	inclusion
Adequate eating habits by kids	-->	Adequate eating habits by family	inclusion
Staple production	-->	Deforestation	inclusion
Adequate eating habits by kids	-->	Food purchase by school	inclusion
Staple production	-->	Food affordability	inclusion
Fruit and vegetable production	-->	Food affordability	inclusion
Livestock production	-->	Food affordability	inclusion
Food affordability	-->	Food purchase by family	inclusion
